# “You are not alone”: Connecting through a bereaved parent mentor program for parents whose child died of cancer

**DOI:** 10.1002/cam4.4696

**Published:** 2022-04-01

**Authors:** Michael J. McNeil, Ashley Kiefer, Cameka Woods, Brittany Barnett, Kathryn Berry‐Carter, Lisa Clark, Belinda N. Mandrell, Jennifer Snaman, Erica C. Kaye, Justin N. Baker

**Affiliations:** ^1^ Division of Quality and Life and Palliative Care, Department of Oncology St. Jude Children's Research Hospital Memphis Tennessee USA; ^2^ Department of Global Pediatric Medicine St. Jude Children's Research Hospital Memphis Memphis Tennessee USA; ^3^ Children's Hospital New Orleans New Orleans Louisiana USA; ^4^ Department of Family, Guest and Volunteer Services St. Jude Children's Research Hospital Memphis Memphis Tennessee USA; ^5^ Department of Pediatrics, Division of Nursing Research St. Jude Children's Research Hospital Memphis Memphis Tennessee USA; ^6^ Department of Psychosocial Oncology and Palliative Care Dana‐Farber Cancer Institute Boston Massachusetts USA; ^7^ Department of Pediatrics Boston Children's Hospital Boston Massachusetts USA

**Keywords:** bereavement, bereaved parents, grief, pediatric palliative care, peer‐to‐peer mentorship

## Abstract

**Background:**

Bereavement after the death of a child is devastating and associated with worse physical and psychosocial well‐being in parents. Evidence suggests that parents desire and benefit from support provided by other bereaved parents. To foster this peer support, an institutional peer‐to‐peer mentorship program for bereaved parents was established, through which trained bereaved parent mentors offer support for newly bereaved parents.

**Methods:**

Using a retrospective cohort design, we describe the characteristics of participants of the Bereaved Parent Mentorship program. Trained bereaved parent mentors documented encounters with newly bereaved parent mentees using a secure internet‐based form. Mentors summarized each encounter including any concerns or need for professional psychosocial support. Descriptive statistics were used to describe mentor and mentee characteristics; free text from encounter summaries was qualitatively analyzed using content analysis.

**Results:**

A total of 1368 documented encounters occurred between 150 mentees and 39 mentors from January 1, 2014 to February 29, 2020. Only seven encounters (0.5%) were flagged as serious concern necessitating professional psychosocial support. Four key themes in the encounters between mentors and mentees emerged, including: descriptions of the grief experience, ways in which a mentor supported their mentee, challenges the mentor experienced in supporting the mentee, and personal benefit gained by the mentor from supporting their mentee.

**Conclusion:**

This structured Bereaved Parent Mentorship program fostered rich interactions between bereaved parent participants, with very few encounters requiring professional assistance. Future research will assess the impact of bereaved mentor programs on resilience and psychosocial, physical, and functional well‐being of parents.

## Graphical abstract

This structured Bereaved Parent Mentorship program for parents whose children died of cancer fostered rich interactions between bereaved parent participants. Very few encounters in this program required professional psychosocial assistance. Future research will assess the impact of bereaved mentor programs on resilience and psychosocial, physical, and functional well‐being of parents.

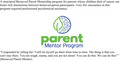

## 1 INTRODUCTION

The death of a child is devastating and provokes intense grief reactions in bereaved parents.^1^, ^2^ After a child's death, parents are more likely to have significant psychiatric sequelae,^3^, ^4^, ^5^ increased risks of cardiac morbidity,^6^ poorer health‐related quality of life,^7^, ^8^ and even increased mortality compared to non‐bereaved parents.^9^

A range of interventions for bereaved parents exist including pharmacotherapy, counseling, psychotherapy, and systems‐oriented interventions.^10^, ^11^, ^12^, ^13^, ^14^ However, studies involving bereaved parents identified additional sources of support requested by parents and families.^15^, ^16^, ^17^ These services included structured bereavement support, mailings, memorial events, and support groups. These services are often desired because they provide an opportunity to connect with other bereaved parents. Parents find interactions with other bereaved parents as helpful because it offers opportunities for shared experience, normalization of grief, and helpful insight into the future.^15^, ^16^, ^17^

An additional intervention that has potential benefits for parents is that of formal, structured peer support.^18^, ^19^, ^20^ For example, a trained parent mentor can provide anticipatory guidance and practical advice as they share their experience of navigating a child's illness and end of life, as well as provide support during bereavement.^21^, ^22^ Other parental peer support networks exist, both for parents of children receiving active treatment and those who have experienced the death of a child.^18^, ^23^, ^24^, ^25^, ^26^, ^27^ While these studies describe the program development and execution, there remains a poor understanding of who participates and the content and impact of the peer‐to‐peer interactions. Additionally, hospitals and institutions may express concerns about developing this kind of program due to the training and supervision required, along with the professional support needed for both mentee and mentor.

The Bereaved Parent Mentorship (BPM) program was developed at the request of the institutional Parent Family Advisory Council (PFAC) and provides a structured approach to parent‐to‐parent mentoring for parents whose children died of cancer.^17^ This study aims to describe the characteristics of the mentors and mentees, better understand the components of bereaved parent mentor–mentee relationships within the BPM program and identify potential areas for programmatic improvement.

## 2 METHODS

This study was approved by the Institutional Review Board at St. Jude Children's Research Hospital (SJ‐BPMP [FWA00004775]; approval date: 06/18/2020).

### 2.1 Bereaved Parent Mentorship program

The BPM program was first launched in 2014 and comprises multi‐disciplinary team members (palliative care physicians, psychologists, social workers, chaplains, volunteer services, patient family‐centered care, and parents). Parent mentors participate in the BPM program through self‐nomination, referral by members of the psychosocial staff and medical team, or by intake through the Patient Family Centered Care (PFCC) program staff. Potential parent mentors must be at least 2 years from the death of their child, complete a comprehensive application and criminal background check, and interview with PFCC staff. Approved mentors also complete web‐based training modules outlining mentoring skills, role of mentors, and mentor self‐care. Additionally, mentors are trained on situations and encounters with their mentee that warrant additional professional support such as a mentee verbalizing thoughts of self‐harm or desire to harm others. Following completion of the modules, mentors are required to attend an in person or virtual training taught by multidisciplinary psychosocial staff. Details on this program and training have been previously published.^27^

Newly bereaved parents receive information on the program from psychosocial team members prior to the death of their child and/or through regular bereavement support mailings, which include a list of resources and materials for parents after the death of their child. Parents may either self‐refer or be referred by their psychosocial or medical team. Parent mentees are then matched with a parent mentor based on child's age and cancer diagnosis, family structure (single vs. partnered) and/or specific needs or concerns of the parent.^27^ The initial encounter between the mentor–mentee dyad is initiated by the mentor via phone call or text message and mentors make contact about one time per month or as needed to provide support for the mentee. The formal mentorship period is for 15 months but can be extended up to another 12 months if requested/recommended.^27^

### 2.2 Data collection

Every encounter, including the initial contact, is documented by the mentor in an electronic form and stored in a protected SharePoint site (Figure S1). The encounter form is separate from the medical record and is only accessible by members of the psychosocial team. The form includes mentee name, date of the encounter, type of encounter (phone call, text, in person, other), amount of time spent, any referrals made (medical team, social work, spiritual care, etc.) and a free text description of the encounter. Mentors can mark the encounter with a “pink flag” denoting minor issues such as excessive parental worry, financial concerns, or need for additional resources, or a “red flag” indicating more urgent concerns such as complaints toward staff or suicidal ideation. The encounter forms are closely monitored by staff. A red flag is immediately referred to a member of the hospitals' psychosocial staff and addressed. The mentors are given the opportunity to meet regularly and have close communication with the mentor program leadership team to discuss all encounters and any concerns including pink flags.^27^ All encounters between mentors and mentees were obtained from the BPM program SharePoint website between January 1, 2014 and February 29, 2020. Demographic data not collected from SharePoint was obtained from the patient's medical record.

### 2.3 Codebook development

Free text responses of mentor documentation were assessed using memo‐writing followed by inductive codebook generation to identify the concepts and codes that described the interactions and relationships between the mentor and mentee.^28^ Novel codes were developed from the content of the mentor descriptions of the mentor–mentee interactions. After codebook development, qualitative research analysts (MJM, AK) independently piloted the codebook across several encounters to ensure consistent application of codes. The research team met regularly to resolve differences in code application and modify the codebook, when necessary, to ensure consistency, reliability, and credibility in coding.^29^

### 2.4 Analyses

Demographic features of the study population were analyzed by descriptive statistics. Encounters were analyzed through inductive content analysis.^30^ After developing the codebook, all the encounters were coded independently by two coders (MJM, AK) using the mixed methods data analysis software system MAXQDA.^31^ The research team then met regularly to review coding variances with third‐party adjudication (JNB, CW) as needed to achieve consensus. Broad themes were then developed inductively from the encounters, focusing particularly on the relationship between the mentor and mentee. The research team (MJM, AK, JNB, CW) met regularly to ensure consistent thematic development and analysis. The COREQ checklist was used in the development and reporting of our qualitative data.^32^

## 3 RESULTS

### 3.1 Demographics

During the program period, 150 mentees partnered with 39 parent mentors (Table 1). Most mentees and mentors identified as white (69% and 95%), non‐Hispanic (93% and 95%), mothers (80% and 69%), and married (65% and 92%). Central nervous system tumors were the most common malignancy in children for both mentees and mentors (38% and 56%, respectively). Age of death for the mentees' children was evenly distributed from ages 0 to 20, (mean = 11.1 years) with 9% having died at age ≥21 years.

**TABLE 1:** Demographics

Bereaved parent mentees	*n* (%)
Relationship to child	
Mother	120 (80%)
Father	25 (17%)
Other	5 (3%)
Marital status	
Married	97 (65%)
Divorced	9 (6%)
Single	28 (19%)
Widowed	2 (1%)
Unknown	14(9%)
Child's race	
White	98 (69%)
African American	35 (25%)
Asian American	1 (1%)
Native American/Pacific Islander	1 (1%)
Other	7 (4%)
Child's ethnicity	
Hispanic	10 (7%)
Non‐hispanic	132 (93%)
Child's diagnosis	
Hematologic malignancy	41(29%)
Central nervous system tumor	54(38%)
Solid tumors	45 (32%)
Other	2 (1%)
Participation in phase I clinical trial	
Yes	35 (25%)
No	107 (75%)
Child's age at death	
0–5 years	36 (25%)
6–10 years	30 (21%)
11–15 years	34 (24%)
16–20 years	29 (21%)
21+ years	13 (9%)
Bereaved parent mentors	*n* (%)
Gender	
Male	12 (31%)
Female	27 (69%)
Marital status	
Married	36 (92%)
Divorced	2 (5%)
Single	1 (3%)
Race	
White	37 (95%)
African American	2 (5%)
Ethnicity	
Hispanic	2 (5%)
Non‐hispanic	37 (95%)
Child's diagnosis	
Hematologic Malignancy	10 (26%)
Central Nervous System Tumor	22 (56%)
Solid Tumors	6 (15%)
Other	1 (3%)
Time since child's death	
5–8 years	24 (62%)
9–12 years	13 (33%)
13+ years	2 (5%)

### 3.2 Encounters

We examined 1359 encounters between mentors and mentees. Median number of encounters between mentor–mentee dyads was 7 (range: 1–67 encounters). Most encounters were done via text message (57%), followed by telephone calls (19%). Email, cards, and in‐person interactions were less common (5%, 2%, and 2%, respectively), while 15% did not specify. Contact was typically initiated by the mentor (95%); 61% of mentor‐initiated encounters received a response from the mentee. Only 46 (3.5%) encounters were flagged for non‐urgent follow‐up (pink flag) and only seven (0.5%) encounters were flagged for urgent follow‐up (red flag) (Table 2).

**TABLE 2:** Encounter information

Type of encounter	*n* (%)
In person	21 (2%)
Phone Call	262 (19%)
Text	778 (57%)
Email	61 (5%)
Card	28 (2%)
Didn't specify	209 (15%)
Mentee response	
Yes	823 (61%)
No	312 (23%)
Did not specify	224 (16%)
Who initiates contact	
Mentor	1290 (95%)
Mentee	69 (5%)
Flagged for follow‐up	
No	1306 (96%)
Yes (pink flag)	46 (3.5%)
Yes (red flag)	7 (0.5%)

### 3.3 Themes

We identified four key themes in the free text mentor‐mentee encounter summaries: (1) descriptions of grief, (2) ways in which a mentor supported their mentee, (3) challenges experienced by the mentor in supporting the mentee, and (4) personal benefit and meaning gained by the mentor because of supporting their mentee. (Table 3).

**TABLE 3:** Codebook, definitions, and example quotes

Theme/sub‐theme	Code	Definition	Quotes
Descriptions of grief			
	Grief as a journey	Mentor or mentee describing the grief and bereavement experience as a fluctuating or never‐ending process	We spoke about learning how to carry this pain, having to shift the weight, and do different things to adjust to life without (daughter). We also spoke about how the pain and weight of her death will always be with her, but as time goes by how she (mother) will learn the adjustments she has to make to carry that pain.
	Unique nature of grief	Validating that grief is an individualized experience for each bereaved family member	She said that her husband and her mother still get sad when she talks about (daughter). She finds comfort in talking about her. I told her that it is normal for spouses to grieve differently and shared my personal experience with that. I suggested that in time this may change for her husband.
Mentor support			
	Advice/guidance/encouragement	Mentor provides counseling and support to address challenges experienced by bereaved parents	Advice: I suggested maybe taking time to make a memory jar. When they think of something about (son) they write it down and place it in a jar and they can keep adding to it. Over time they want to take the memories out and read them when they are missing him.
			Anticipatory Guidance: (Daughter's) birthday is coming up in 2 weeks and the first anniversary of her death in October. We talked about what those dates might be like for her and how she might get through them.
			Encouragement: I responded by telling her “I still let myself go there from time to time. The thing is that you cannot stay there. You are tough, mama, and you are not alone! You can do this. We can do this! And if you feel like you need a little extra help then please reach out to our Bereavement Coordinator.”
	Praise	Mentor commends and compliments mentee for actions, behaviors, self‐awareness, etc. during grief process	Spoke at length that this was normal, and she should be proud of herself for doing something productive every day.
	Normalization	Mentor normalizes mentee experience with that of other bereaved parents or in general to the bereavement experience	She also shared that she had a bad few days recently but that it had passed and she was feeling better. I shared that was very normal and that I still have bad days but yes, they do pass. I shared that sometimes you know what triggers these emotions and sometimes you do not
	Sharing personal experiences	Mentor describes personal experience with grief as way of supporting mentee	I spoke about how I talk with (mentor's son) about how I continue to love him even though I get sad about (mentor's son). She noticed similarities between (mentor's son) and (brother). They are both very focused, good in school, overly organized just to name a few. I told her I felt it was something they could control when they were in such an uncontrollable situation for so long. I told her (mentor's son) was just now starting to mention a few things randomly about how he feels like he dealt with (mentor's son's) entire journey. I assured her I would share anything I learned from (mentor's son).
	Bereavement resources	Mentor recommends, informs, or seeks out resources for mentee including support groups, grief counseling, online websites, books, etc.	I asked if she had any counseling options and she commented they were too expensive. Is there anything St. Jude can do to research her area for affordable options. I also asked if she was affiliated with a church, and she said yes. I recommended that she call the pastor and see if he could offer counseling assistance or options at no charge. I'm going to research to see if there is a Grief Share program in her area. If so, I will send her the information.
	Thinking of you	Mentor shares that they are thinking of mentee and/or reiterates their availability whenever needed	She asked permission, or if it was ok, to send things that are depressing her, and seemed concerned that she was going to make me have a bad day. I let her know that this was the place to vent and to share.
Mentor challenges	Feeling ill‐equipped	Mentor expresses apprehension/doubt regarding their ability to support mentee	I'm not sure I am helping her as much as I may be hindering her.
	Still grieving	Mentor continues to grieve the loss of their own child	We also discussed it not ever being easier for me, and not time healing the wound for myself, but knowing that I will carry (mentor's daughter) with me forever. Sometimes the weight is so heavy and other times it is noticeable, but at the same time I realize I have adjusted somehow to manage it better.
Mentee to mentor	Therapeutic benefit	Mentee expresses the value added by having mentor support/ advice	She expressed her gratitude to me and to the mentor program. She said that it has really helped her. She is even thinking about possibly becoming a mentor herself in the future.
	Mentee supporting mentor	Mentee provides encouragement and support to mentor	She asked about (mentor's son's) death anniversary coming up in June which I thought was really sweet. She offered to be a support to ME when it got close to that time.

### 3.4 Descriptions of grief

In conversations between mentors and mentees, bereaved parents often described their grief experience. A common description was that grief is a never‐ending journey. One mentor summarized the conversation with her mentee:

“She expressed how time has gone by both so incredibly slow and fast all at once. She hangs on to the good memories but expresses how she wishes she could give him a hug. I responded with understanding the wave of emotions even as time goes on without our precious children. I told her to be patient with her grief as it is a journey, we as parents will be on forever.”

Another common description was the unique nature of grief and that each parent must navigate their own individual bereavement experience. One mentor reminded her mentee,

“…everyone grieves differently and that it is good that she is searching out what might help her get through this unavoidable process.”

### 3.5 Mentor supporting mentee

Mentors provided support to their mentees in a variety of ways. Often, the mentor provided practical advice, anticipatory guidance, or encouragement to the mentee. One mentee described how difficult it was to be unable to visit their child's gravesite several hours away. The mentor counseled the mentee with this advice:

“Ask[ed] if she had any area in her house or yard that was a special place for (daughter). Talked about putting a steppingstone, angel, plant or something in an area that would be (daughter's) special area. That way when she couldn't get to cemetery, she still had an area that special to her.”

Another mentor provided anticipatory guidance about the holidays to their mentee, writing:

“I told her the firsts are always difficult and that she needs to do what works for her, her husband and (sister). I stated that whether she honors traditions they previously had or create new ones, they need to do what feels right to them. Told her not to worry about pleasing others.”

In addition to providing advice and guidance to their mentees, mentors also encouraged them to keep going even when things were difficult. They offered praise and encouraged them during difficult moments. One mentor stated:

“I still let myself go there from time to time. The thing is that you can't stay there. You are tough, mama, and you are not alone! You can do this. We can do this!”

Another praised their mentee for starting a foundation in their child's name, writing:

“Told her I thought it is a wonderful thing she is doing and what a positive way to carry on (daughter's) name and legacy and that she should be very proud of her efforts.”

Parent mentors also regularly normalized the bereavement experience of the mentee. One mentor described the conversation in which the pair discussed the challenge of feeling their child's absence after the child's death:

“We spoke of a disconnect between the heart, mind, and reality. She knows (her son) is not here, but actually admitting that is hard. Your mind knows and your heart knows, but they are not always on the same page and that is normal.”

Often, mentors shared their own personal experience to normalize the mentee's feelings or experience. These shared experiences explored the breadth and depth of bereavement and allowed the mentee to reflect on their own experience. One mentor described the stress they experienced during the first Christmas after the death of their child, sharing:

“We discussed this being her first Christmas without (her son). I shared with her how my first Christmas without (my son) was stressful because I was worried about making everyone sad around me because I was sad, but I finally gave myself permission to feel what I was going to feel that day.”

Several parent mentors also sought out and provided education regarding certain supportive resources that they utilized and/or thought might be beneficial to the mentees. This included local support groups or counseling, annual remembrance events or activities at the hospital, and bereavement support resources (i.e., books, websites, etc.). One mentor offered to re‐read supportive books so the pair could discuss them together.

The most common support offered by mentors was an expression of solidarity and availability. One mentor stated:

“I followed up with a text to (grandmother) after hearing of the passing of (grandson). I let her know I understood her pain and that she was not alone and I'm here for her if/when she needs me.”

Many parent mentors quoted the mentees from their encounters as they described the benefit they received from participating in the parent mentor program. One parent mentee told the parent mentor that each time she received a text from her mentor that it: “felt like a hug.” Another parent mentor described her mentee's gratitude by stating:

“(Mother) replied thanking me for helping her through this past almost 2 years. She said that our texting always cheered her up and let her feel like she was not alone. She also asked if we could still keep in touch because she did not want to say goodbye.”

### 3.6 Mentor challenges

Bereaved parent mentors identified several challenges related to their mentorship role. Some parents verbalized feeling ill‐equipped to address their mentee's concerns or provide needed support. One mentor voiced frustration, writing:

“At this point I really don't think there is anything I can do for her. She does not tend to act on any advice given. I will continue to listen and hopefully that's enough.”

Another mentor expressed concern that he/she could not meet the mentee's needs:

“She doesn't seem to be a ‘talker,’ I feel like she wants me to tell her what to do to make things easier and I'm struggling with what to say to her (guess I'm a better listener than advice‐giver).”

Finally, parent mentors recognized that they are still grieving the death of their own children while also providing support to a newly bereaved parent. One mentor stated:

“Explained it was a rough week for me and she thanked me for telling her that. Spoke about how even though it had been 3 years I still have rough times. Believe it's best to acknowledge the emotions and feel it rather than try to hide it.”

### 3.7 Benefits for the mentor

Mentors recognized that the therapeutic benefit of the BPM program was not unidirectional. Some mentors described the benefit they received by sharing their child's stories and strength they feel by providing support for their mentees. Other mentors described how the mentorship was not one‐sided. One mentor stated,

“I told her she does have a lot of strength and that she is always an inspiration to me. I explained that in some ways we are mentoring each other. I wished her a wonderful weekend”

In several instances as well, a mentee reached out to their mentor to offer support on what they knew would be a difficult day, such as the anniversary of the mentor's child's death. One mentor stated,

“She asked about (mentor's son's) death anniversary coming up in June which I thought was really sweet. She offered to be a support to me when it got close to that time.”

## 4 DISCUSSION

This is the first analysis of a parent‐initiated, parent‐centered, institute‐supported bereaved parent mentorship program. Quantitative analysis demonstrated a large number of mentor–mentee connections, resulting in mutual benefit. Although very few encounters occurred requiring professional follow‐up, parents did identify barriers to providing needed support for their mentees.

Parent mentors provided support to their mentees by finding similarities between their grief journeys in order to “normalize” the mentees' grief while also validating the unique nature of the bereavement experience.^33^, ^34^, ^35^ This ability to relate to their own personal grief allowed the parent mentor to provide advice, encouragement, and anticipatory guidance that many medical professionals may not be able to provide. Mentors also received support from their mentees and were encouraged to talk about their child. Sharing their child's story and using this to support others, may help parents to find meaning in their loss.^36^, ^37^, ^38^ This support may be influenced by the connection that the parents have with the institution that cared for their child. Parents often have a strong attachment to the institution and healthcare professionals involved in their child's care and this by extension may influence the interaction between parent mentors whose children were also cared at the same institution.^16^, ^17^ Further research will be needed to better understand how serving as a bereaved parent mentor affects the mentor's own grief experience.

Importantly, very few encounters required additional professional support or follow‐up. Many institutions may fear developing a peer‐mentoring program due to the concern that it may result in undue harm to both the mentee and mentor. However, of the over 1300 encounters between our dyads, only 46 (3.5%) encounters were flagged for non‐urgent follow‐up (pink flag) and only 7 (0.5%) of them were flagged for urgent follow‐up (red flag). With initial investment in training and regular debriefing of mentors, this program was able to provide support to many more parents than if it were run by healthcare professionals and hospital staff alone. Moreover, we found that both mentors and mentees benefited from participation.

This study has several limitations. First, there is a selection bias in studying the experiences of volunteers who self‐selected into the program. This self‐referral may influence the mentors' interactions with mentees in addition to their interpretation of these interactions. Second, these data are specifically from parents whose child died of cancer at a single tertiary care institution and may not reflect the experiences of bereaved parents in other settings. Additionally, most of the participating mentors and mentees were white, non‐Hispanic mothers and most mentors were married. These observations will be important to thoughtfully approach future projects to gain perspective and hopefully provide support to both fathers and underrepresented communities in the future.^39^, ^40^, ^41^ Also, there was a wide range in the number of encounters between parent dyads (1–67). This range occurred due to the time of participation in the program, along with the needs of the parent mentee. For example, the dyad with 67 encounters had several brief check‐ins and also extended her program for an additional 12 months so as to give the mentee more time with support after the death of their child. For other dyads they began meeting during late 2019 or early 2020 toward the end of our evaluation period which limited the number of potential encounters per dyad captured in our data. Finally, data were collected retrospectively from the mentors' perspectives and summaries of encounters. Future projects should prospectively assess the experience of both mentors and mentees for formal qualitative and quantitative analysis to better understand the effect of the BPM program on participants' grief and psychological and physical well‐being after the death of a child.

The death of a child is a uniquely devastating experience. Many parents may feel isolated, as few can understand the depth of their loss. This parent‐initiated, parent‐run bereaved parent mentorship program provides a distinct, supportive experience by pairing newly bereaved parents with a peer mentor who understands their grief in a way that healthcare professionals, friends, and family may not. This qualitative analysis demonstrated the depth of these interactions between mentor and mentee, supporting expansion of this program and encouraging other institutions to start similar programs. Future research should further investigate the therapeutic benefit for both mentor and mentee and when compared to other formalized peer dynamics such as larger support groups.

## CONFLICT OF INTEREST

All authors declare no conflict of interest.

## AUTHOR CONTRIBUTIONS

MJM, AK, ECK, and JNB developed the idea. MJM, AK, CW, ECK, and JNB verified the data. MJM, AK, and CW did the data analysis. MJM and AK drafted the manuscript and prepared the tables and figures. All authors contributed to the interpretation of the findings, the editing of the article, and the approval of the final submitted version. All authors had full access to all the data in the study and accept responsibility for the decision to submit for publication.

## ETHICS STATEMENT

This study was approved by the Institutional Review Board at St. Jude Children's Research Hospital [SJ‐BPMP (FWA00004775); approval date: 06/18/2020].

## Supporting information




Figure S1

## Data Availability

Data available on request due to privacy/ethical restrictions
