# Supplementary figures and images for: “You are not alone”: Connecting through a bereaved parent mentor program for parents whose child died of cancer
Source: Cancer Med. 2022 Apr 1;11(17):3332–41. doi: 10.1002/cam4.4696 (PMC9468435; doi:10.1002/cam4.4696)

**Appendix B**


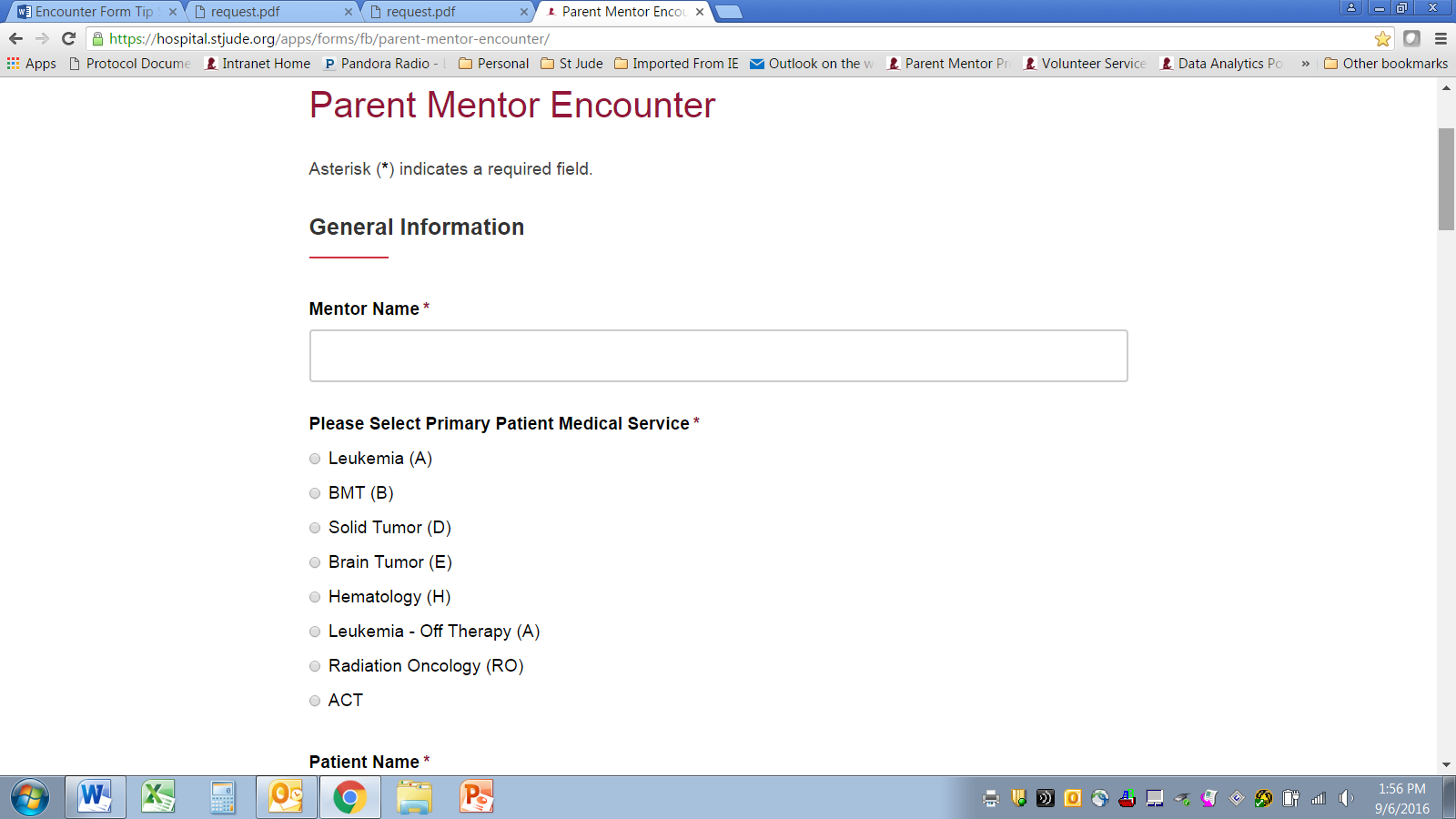


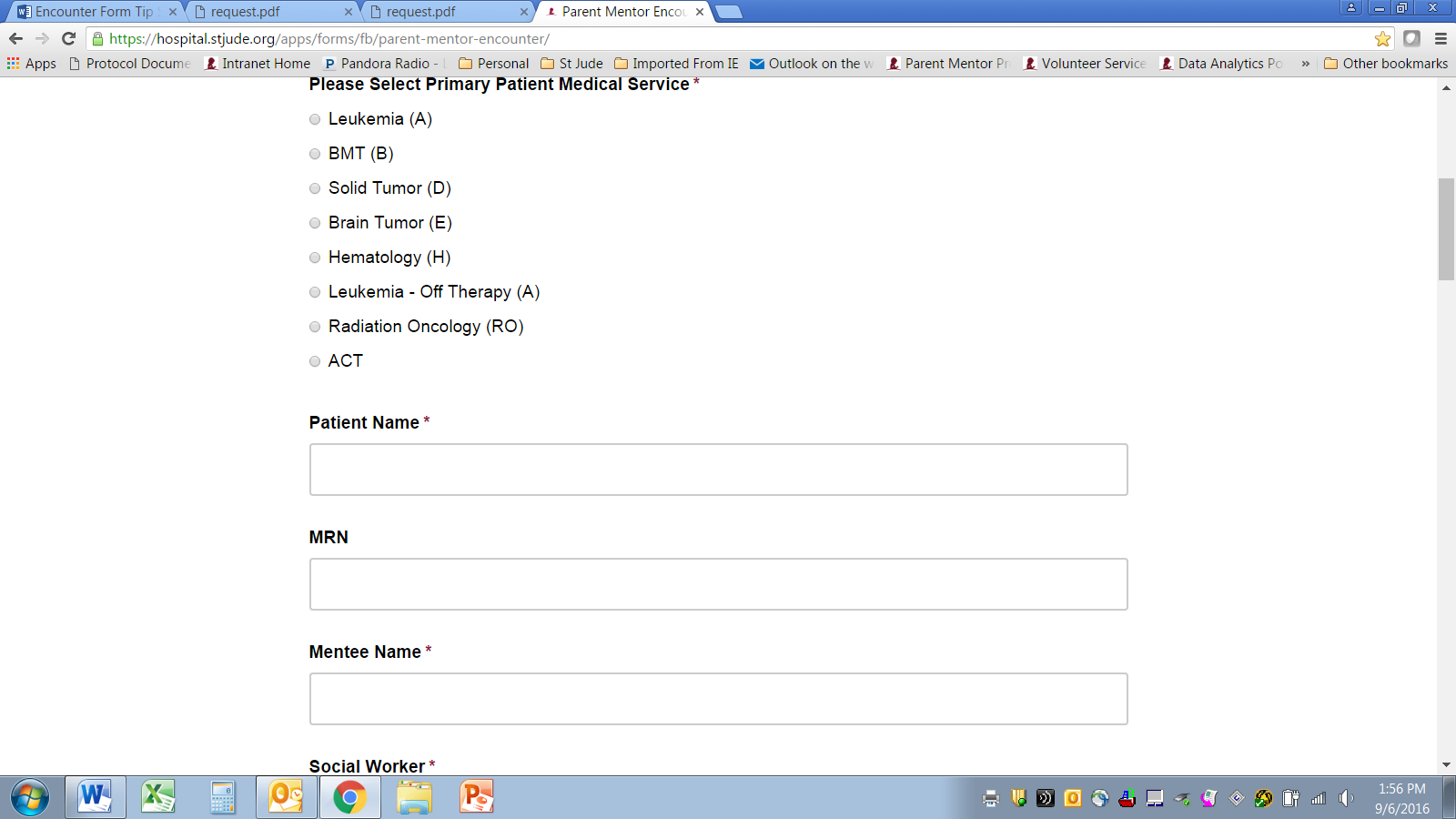

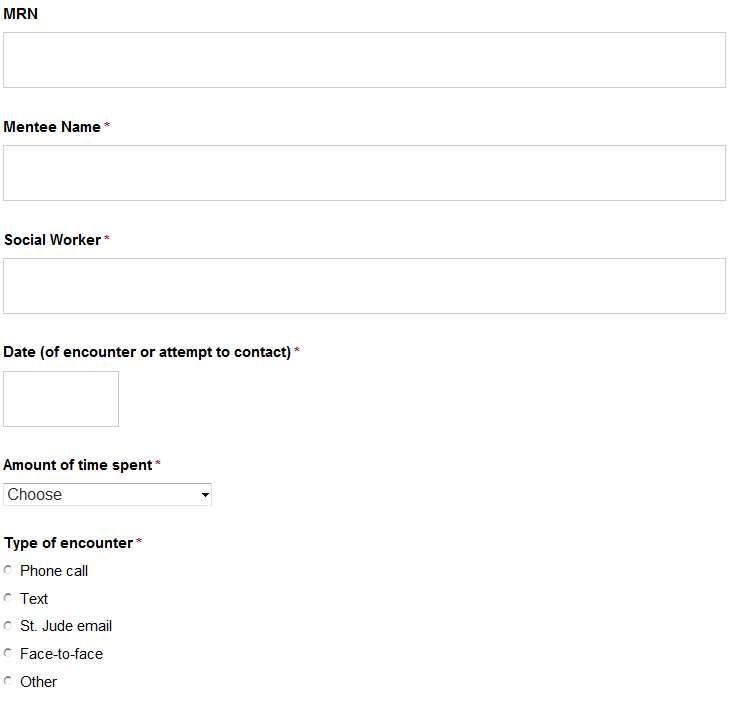


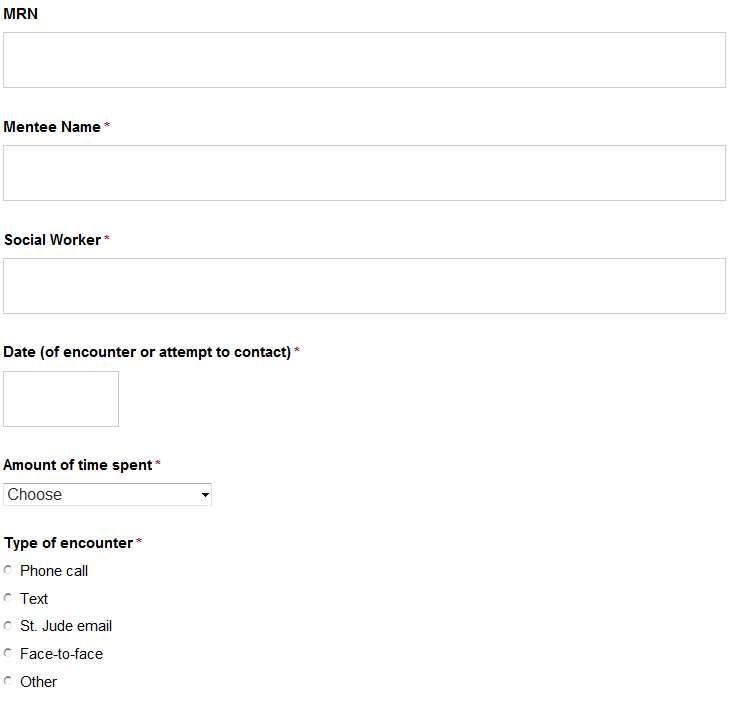


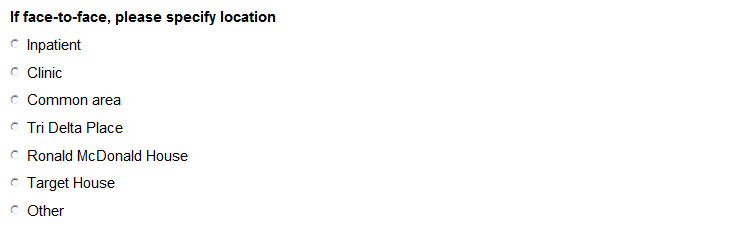


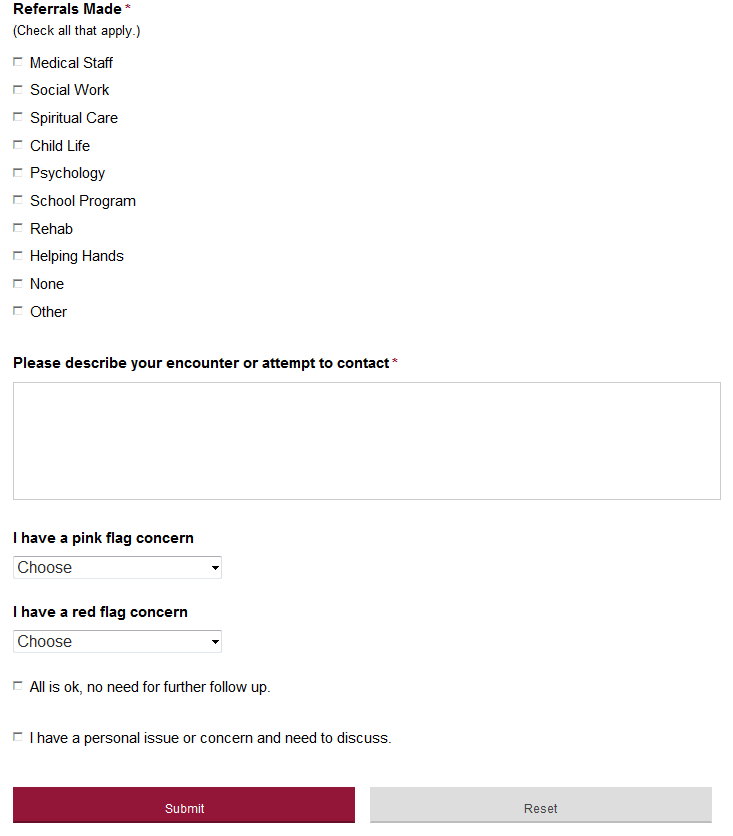

Supplement: Supplementary file 1 — Figure S1 [file CAM4-11-3332-s001.docx]
